# Case Report: Maintenance of Desensitization to Nebulized Colomycin Over 10 Years

**DOI:** 10.3389/fped.2021.663228

**Published:** 2021-04-01

**Authors:** Justyna Sieber, Sabine Renner, Andrea Lakatos-Krepcik, Zsolt Szépfalusi

**Affiliations:** ^1^Division of Pediatric Pulmonology, Allergy and Endocrinology, Departement of Pediatrics and Adolescent Medicine, Comprehensive Center of Pediatrics, Medical University of Vienna, Vienna, Austria; ^2^Department of Clinical Immunology, Wroclaw Medical University, Wroclaw, Poland; ^3^Department of Pulmonology, Klinik Hietzing, Vienna, Austria

**Keywords:** hypersensitivity, desensitization, cystic fibrosis, case report, colomycin, drug tolerance, drug allergy

## Abstract

Drug desensitization can be achieved successfully by gradual drug dose increases in different protocols. Most protocols are designed to obtain temporal tolerance. The data on long-term maintenance of drug tolerance is scarce. Based on an IgE-mediated colomycin allergy we describe the maintenance of drug tolerance to nebulized drug for the period of 10 years in a 15-year-old cystic fibrosis patient, proceeded by successful rush intravenous desensitization protocol. The mechanism of drug tolerance is largely speculative; however, long-term maintenance of it seems achievable by continuous local drug application.

## Introduction

Adverse drug reactions can be considered as important public health problem (1). Patients with Cystic Fibrosis represent a defined risk group for drug allergies (2–4). Desensitization protocols have been successfully developed to prevent from withholding of first line therapy in allergic patients. The principle of this approach is to administrate fractional aliquots of the total therapeutic dose and alter the patient's immune response to a drug. Most of described protocols achieve that goal through oral, intravenous or subcutaneous routes. Thus, many routes seem to be feasible. Starting dose, number of steps and dosing frequency differ between available protocols (5–7). A 12-step standardized intravenous (IV) protocol has been developed and used successfully for immediate hypersensitivity reactions to a variety of drugs (5) In fact, more rush or more prolonged protocols can be successfully used. Nevertheless, drug tolerance used to be temporary and the tolerant state can be lost as soon as after 24 h after discontinuation of the therapy. With recurrent need of antibiotic treatments desensitization procedures have to be repeated. These can be a relevant clinical problem in the management of Cystic Fibrosis, as recurrent antibiotic and continuous or alternate inhaled antibiotic therapy became standard of care, mostly due to chronic Pseudomonas aeruginosa colonization (8). In addition, these pathogens may develop resistances to the narrow group of antibiotic sources. Alternative drugs are rare and the new treatment approaches are warranted. Thus, we describe the successful maintenance of drug tolerance state to colomycin for the period of 10 years in a Cystic Fibrosis allergic patient. This was achieved by daily inhalation of nebulized drug after a successful IV desensitization procedure.

## Case Description

**Figure 1:**
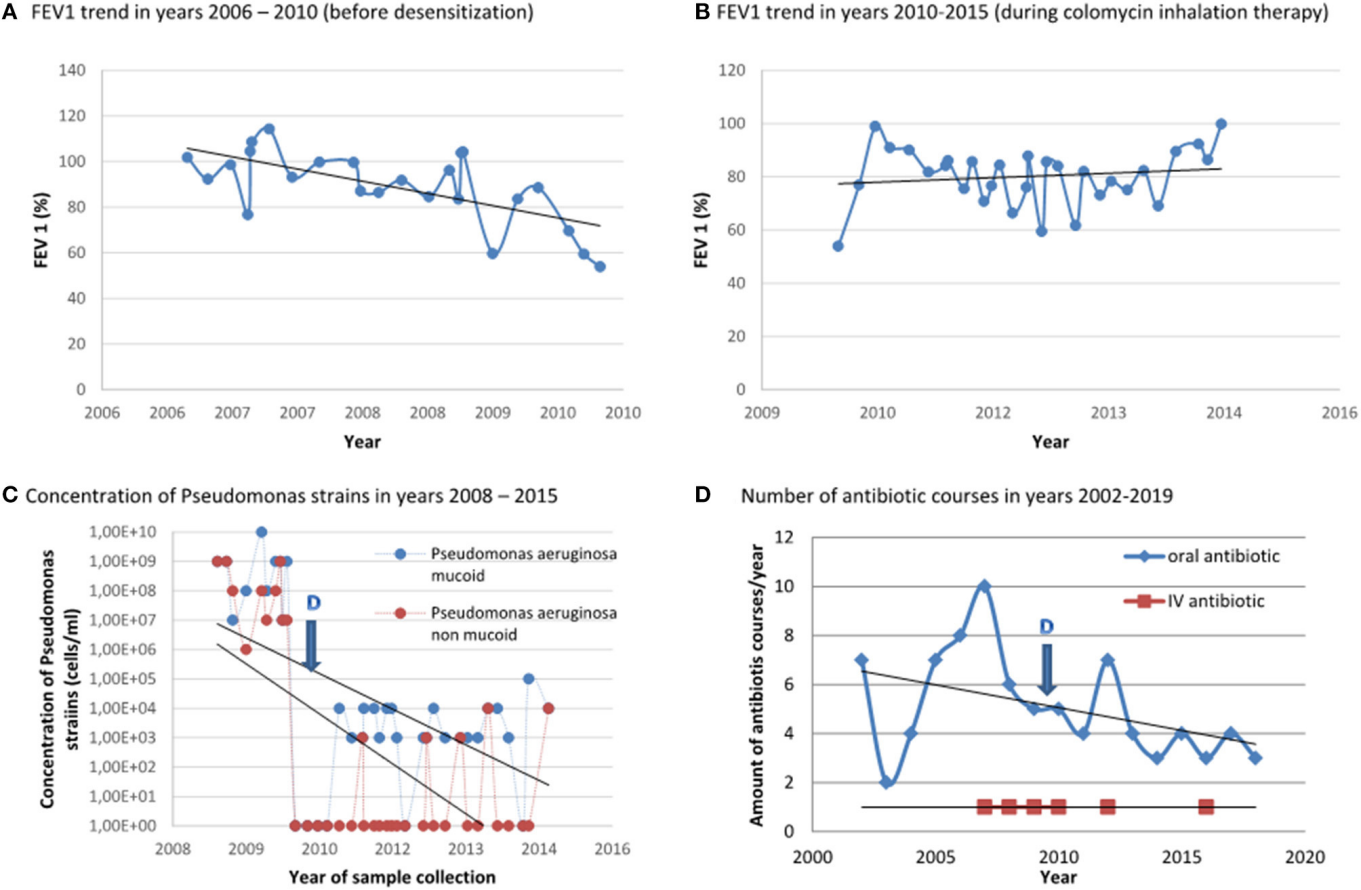
Clinical course of the patient before desensitization and under long-term therapy with colomycin. **(A)** FEV1 trend before desensitization with colomycin, years 2006–2010. **(B)** FEV1 trend after desensitization with colomycin and under regular, twice daily nebulized colomycin therapy, years 2010–2015. **(C)** Concentration of Pseudomonas strains in years 2008–2015–before and after desensitization. **(D)** Number of antibiotic courses in years 2002–2019-before and after desensitization. D–Desensitization: 06.2010.

We report the case of a 15-year-old girl with Cystic Fibrosis (dF508/dF508), with first Pseudomonas aeruginosa infection at 1 year of age, and chronic colonization at the age of 5 years. The standard first line therapy and in consequence eradication of the bacteria was impeded by a hyperresponsiveness of the bronchial airway to nebulized tobramycin and colomycin, regardless of pre-medication with bronchodilator and corticosteroids. The patient was treated regularly with oral antibiotic courses (ciprofloxacin) and the attempts for aerosol administration of anti-pseudomonas antibiotics were repeated. The initial clinical course was stable, with no acute exacerbation. However, the lung function was deteriorating continuously with time. At the age of 12 years the girl was experiencing deterioration of clinical condition with increased need for oral antimicrobials and acute exacerbation resulting in hospital admissions for I.V. treatment. During one of the hospitalizations, intravenous antibiotic therapy with tobramycin and ceftazidime was initiated. Due to new microbiological findings revealing resistance of Pseudomonas aeruginosa to tobramycin, the antibiotic therapy was switched to ceftazidime, flucloxacillin and colomycin, all given intravenously. Minutes after the initiation of the colomycin infusion the girl reacted with generalized urticaria, dyspnea, tachycardia and hypotension. The infusion was stopped and steroids and antihistamines were applied. The patient recovered and the antibiotic therapy was changed and well-tolerated. The colomycin hypersensitivity was confirmed with positive prick-test (7 mm) and clinical significant FEV1 deterioration (>20 %) after inhalation with colomycin. Since then, colomycin was avoided. Further clinical course showed progressive lung deterioration. The microbiological findings revealed repeated detection of Pseudomonas aeruginosa up to 10^8^ cells/ml. The patient was treated regularly with oral antibiotics. Attempts for nebulized tobramycin therapy were tried again, but was not tolerated by the patient. Repeated IV therapies were resulting only in temporary clinical improvement. At the age of 15 years, due to clinical signs of pulmonary exacerbation, the girl was again hospitalized. The lung function revealed FEV1 54% predicted, FVC 75% predicted, the microbiological findings showed two strains of Pseudomonas aeruginosa with resistance to tobramycin and sensitive to colomycin. Due to lack of alternative therapy, colomycin desensitization was initiated. The patient and her legal representatives gave an informed consent for desensitization procedure. At first, a 4-step intravenous desensitization protocol established in our center was applied (Table 1). The target dose of colomycin was 1,000,000 U. Pre-medication with methylprednisolone 1 mg/kg was used. During the desensitization procedure mild symptoms of a rush and restlessness were observed. The symptoms improved without medication and the procedure was continued without modification. After successful desensitization, the intravenous course of colomycin 3 × 1,000,000 IU/day was continued for 14 days without any adverse events. The clinical condition of the patient and the lung function improved significantly. On day 15 the therapy was switched to nebulized colomycin 2 × 1,000,000 IU and was continued for the next 2 days in inpatient setting. The inhalation treatment was well-tolerated, and thus the girl was discharged with the recommendation for long-term nebulized therapy in home care (Table 2). The patient and her parents were instructed on the necessity of strict and regular administration of the inhaled drug and the possible consequences of discontinuation. Anaphylaxis-rescue medication was prescribed and management training was performed. This therapy was successfully continued for a period of 10 years without any relevant complication. The clinical condition improved significantly, the lung function trend was stable, the microbiological findings of Pseudomonas aeruginosa were reduced to 10^3^ cells/ml, also the number of oral and intravenous antibiotic courses could be reduced as well as the number of hospital admissions due to pulmonary exacerbations (Figure 1).

**Table 1:** Protocol for intravenous desensitization with colomycin.

**Medication**	**Dilution**	**Units**	**Time of infusion**	**Way of administration**
Colomycin	1/1000	1,000 U	60 min	intravenous
Colomycin	1/100	10,000 U	60 min	intravenous
Colomycin	1/10	100,000 U	60 min	intravenous
Colomycin	9/10	900,000 U	60 min	intravenous

**Table 2:** Protocol for long-term desensitization to nebulized colomycin.

**Step**	**Therapy**	**Duration**	**Units**	**Way of administration**	**Settings**
1.	Colomycin–desensitization	1 day	Cumulative 1,000,000U	intravenous	inpatient
2.	Colomycin–treatment course	14 days	3 × 1,000,000 U/day	intravenous	inpatient
3.	Colomycin–nebulized therapy	2 days	2 × 1,000,000 U/day	inhalation	inpatient
4.	Colomycin–nebulized long-term therapy	10 years	2 × 1,000,000 U/day	inhalation	outpatient

## Discussion

Adverse drug reactions are considered important public health problems, because of their frequent occurrence, occasional severity and impact on the use of medications (1). The prevalence of adverse drug reaction in Cystic Fibrosis (CF) is higher than in general population and is predicted to increase (2, 3). This is due to increased use of high dose intravenous antibiotics, frequent antibiotic courses and improving survival in patients with CF (4). Drug desensitization is a procedure which alters the immune response to the drug and results in temporary tolerance (5–7). The exact mechanisms which allowed that tolerance are not fully elucidated. Actually proposed mechanisms are: alteration in expression of surface receptors on mast cells and basophils (9, 10), generation of IgG blocking immunoglobulins (11) and reduction in cellular signaling in mast cells and basophils (12, 13) and presence of inhibitory receptors (14). The indications for desensitization are limited to these cases, in which no acceptable alternative drugs are available and when the benefits of such procedure outweigh the potential harm (15). Many protocols with different escalation steps for various antibiotics have been described so far (16). Most of published desensitization protocols are designed to obtain temporary desensitization. In case of recurrent need for therapy the desensitization procedure need to be repeated as well. Nevertheless, the potential to maintain the drug-tolerant state after drug desensitization upon chronic daily administration has been suggested especially for oral drugs (penicillin) (11).

The present case is due to an IgE-mediated allergy to colomycin. The clinical history with immediate reaction and generalized symptoms, positive SPT and clinical significant lung function deterioration after inhalation of nebulized colomycin reveal an IgE-mediated origin. Additionally, the patient presented airways hyperreactivity to nebulized tobramycin. At that time, as alternative, nebulized therapy with aztreonam was not available yet.

Antipseudomonal treatment in CF patients is challenging. Eradication upon chronic infection is virtually impossible. No consensus has been reached concerning route, choice of the antibiotic, the dosage and duration of the treatment against Pseudomonas aeruginosa in CF patients. In accordance to European consensus for antibiotic therapy against Pseudomonas aeruginosa the patients can be treated with intravenous, oral, nebulized and combined therapy, using different antibiotics (8). The presented patient was treated regularly with oral antipseudomonal antibiotics, which allowed avoiding frequent hospital admission for IV therapies. Unfortunately, nebulized therapy was impeded due to hyperresponsiveness of the airways. For obvious reason, CF patient prefer oral treatment to intravenous ones. Additionally, there are evidences that oral ciprofloxacin can be as effective against Pseudomonas aeruginosa as an intravenous application (17). Nebulized antimicrobial therapy can increase this effectiveness significantly (18, 19). Thus, upon deteriorating clinical condition, presence of high Pseudomonas concentrations and repeated resistance against alternative drugs, colomycin desensitization was performed. Based on the need of a long-term treatment with an effective medication, a strategy to maintain the drug desensitization by nebulized colomycin was attempted. In fact, there are evidences for susceptibility testing being not predictive for clinical success in the treatment of pulmonary exacerbations in Pseudomonas aeruginosa infection (20). The significance of antibiotic resistance in prolonged nebulized therapy remains unclear at present. Taking into account all clinical data, the choice of colomycin over tobramycin seemed reasonable. Desensitization with colomycin is uncommon. However, there is some evidence for successful IV desensitization with that drug (21). The first evidence for desensitization with nebulized antibiotics (tobramycin) has been described in 1995 (22). Since then, there is sparse evidence of desensitization to nebulized antibiotics, such as tobramycin or aztreonam (23–25). The first evidence of desensitization to nebulized colomycin using incremental doses of nebulized drug was described in 2007 (26). Long-term nebulized therapy with colomycin upon IgE-mediated allergy has not been described so far.

Thus, we report for the first time a successful maintenance of a desensitization state to nebulized colomycin over the period of 10 years. The strength of the reported case is the proven IgE-mechanism of a drug adverse event, the length of drug desensitization state and the co-association of a clinical improvement of the patient under this treatment. Data concerning desensitization to nebulized antibiotic is scarce, just like evidence of long-term maintenance of drug tolerance. The biggest limitation of the presented case is the fact, that the mechanisms of drug tolerance remain unknown. Additionally, the described clinical approach needs very compliant patients, since the time of loss of tolerance after discontinuation of the therapy remains unknown, but is thought to occur rapidly.

## Conclusion

Long-term drug tolerance seems to be achievable by repeated/continuous local drug application. The mechanism of drug desensitization is largely speculative and need to be further elucidated. Combined intravenous desensitization procedure followed by an inhaled long-term maintenance treatment appears feasible and may provide a possible treatment approach in particular cystic fibrosis patients needing many antibiotic treatment courses. As inhaled antibiotic are being increasingly used, standardized desensitization protocols with these drugs might be developed.

## Data Availability Statement

The original contributions generated for the study are included in the article/supplementary material, further inquiries can be directed to the corresponding author.

## Ethics Statement

Ethical review and approval was not required for the study on human participants in accordance with the local legislation and institutional requirements. Written informed consent to participate in this study was provided by the participants' legal guardian/next of kin.

## Author Contributions

All authors contributing to the writing of the manuscript, critical review, and accepted the final version.

## Conflict of Interest

The authors declare that the research was conducted in the absence of any commercial or financial relationships that could be construed as a potential conflict of interest.
